# Advances in brain MRI for CAD-associated cognitive impairment with supplementary perspectives from traditional Chinese medicine

**DOI:** 10.3389/fmed.2026.1770913

**Published:** 2026-03-23

**Authors:** Yuan-Feng Liu, Zhi-Zhuo Guan, Xiu-Yun Zhang

**Affiliations:** 1 Department of Pediatric Massage, Affiliated Hospital of Shandong University of Traditional Chinese Medicine, Jinan, China; 2 Department of Acupuncture and Tuina Therapy, Shandong University of Traditional Chinese Medicine, Jinan, China; 3 Department of Acupuncture, The First Affiliated Hospital of Shandong University of Traditional Chinese Medicine, Jinan, China

**Keywords:** cardiovascular remodeling, cognitive impairment, coronary artery disease, heart-brain axis, multimodal magnetic resonance imaging

## Abstract

Coronary artery disease (CAD) and cognitive impairment (CI) are becoming more and more a public health challenge, and the heart-brain axis theory can describe their correlation. In this review, we highlight recent research and future directions for brain magnetic resonance imaging (MRI) based on CAD. Recent studies show that CAD damages the brain’s white matter microstructure, disrupts brain functional network connectivity, and causes gray matter atrophy through multiple pathological mechanisms, including chronic cerebral hypoperfusion, cerebral small vessel disease, neuroinflammation, and oxidative stress, which in turn cause cognitive decline. Recently, multimodal brain MRI has been used to assess damage to the heart-brain axis. Graph-theoretic analysis and artificial intelligence can be used to learn the relationships between abnormal brain network topology, cardiovascular risk phenotypes, and brain imaging features in CAD patients, and have been proven to be a promising biomarker. Notably, this review is the first to systematically explore brain MRI biomarkers as objective tools for validating the scientific basis of traditional Chinese medicine (TCM) heart-brain theory in CAD-related CI. By elucidating the convergence of TCM holistic concepts with modern neuroimaging, we provide a novel imaging assessment framework to guide the development of integrated TCM-Western medicine strategies for the synergistic treatment of heart and brain.

## 1 Introduction

Coronary artery disease (CAD) and cognitive impairment (CI) are both closely related to aging, and this becomes a significant public health problem (1). Epidemiological analysis indicates that in a healthy population, patients with CAD are highly at risk for all-cause dementia and vascular dementia. Moreover, vascular risk factors like hypertension or diabetes form the major intersection between the two, and they share a pathological origin that has a leading role in both cardiovascular and cerebrovascular diseases (2, 3). In the face of accelerating global population aging, systematically understanding the link between CAD and CI is important and scientifically relevant for the effectiveness of developing integrated heart-brain prevention and treatment, as well as for implementing precision interventions.

Heart-brain axis theory can be used to understand CAD and CI. Heart and brain do not play a game independently; they participate in bidirectional regulation via autonomic, neuroendocrine, hemodynamic, and immune-inflammatory signaling (4, 5). CAD can also lead to insufficient cerebral perfusion, systemic inflammation, and oxidative stress, and also contribute to neuronal damage and cognitive decline (6–9). This new medical idea belongs to the same aspect as TCM. Recently, there has been evidence of the relation between heart and brain axis. Guo et al. found that AF patients had low DTI-ALPS indices and impaired lymphoid system function. This impaired lymphodis is a major cause of cognitive decline caused by AF; AF causes cognitive decline by disrupting the liver’s detoxification system. Restoring sinus rhythm by ablation surgery can restore the active lymphoid System (10). Zheng et al.’s (11) study found that patients with heart failure exhibit lymphatic system dysfunction, and the mean ALPS index is closely related to cardiac and cognitive function. Cardiac injury-related lymphatic system dysfunction in heart failure patients may be associated with cognitive impairment. Some patients with coronary heart disease exhibit reduced function of the glymphatic system, and a decreased diffusion tensor imaging (DTI) ALPS index has been linked to cognitive deterioration. Moreover, brain-directed regulatory approaches, such as transcranial magnetic stimulation, have been shown to exert reciprocal effects on cardiac function. These findings provide additional biological support for the TCM notion that the smooth circulation of qi and blood plays a critical role in maintaining heart – brain homeostasis (12).

Magnetic resonance imaging (MRI) has become a key technique for elucidating cerebral alterations along the heart-brain axis in this field of research. Structural MRI (sMRI) and DTI enable quantitative assessment of brain atrophy and white matter microstructural damage (13, 14). At the same time, arterial spin labeling (ASL) and blood oxygenation level-dependent (BOLD) functional MRI are used to characterize cerebral perfusion and the reorganization of functional connectivity networks, respectively (15). In recent years, MRI image analysis augmented by artificial intelligence has continued to advance: machine learning approaches can reveal patterns of brain atrophy or connectivity associated with distinct cardiovascular risk phenotypes (16); radiomics models hold promise for improving the prediction of CAD related cognitive decline (17); and high dimensional fMRI analytical frameworks designed for spatiotemporal dynamic signals further enhance the depiction of large scale brain network activity and clinical discriminative performance (18). In addition, prospective heart brain synchronized MRI protocols aim to jointly evaluate cardiac microvascular function and cerebral small vessel pathology within a unified spatiotemporal scale, providing a new technical pathway for elucidating the bridging mechanisms between the two systems (19).

Given that CAD related CI involves multi level and cross systemic pathological processes, this article systematically integrates recent research progress based on brain MRI. It summarizes the epidemiological evidence and key pathological mechanisms of CAD induced cognitive impairment, clarifies its unique advantages in identifying heart-brain axis damage, and summarizes a system of quantifiable imaging biomarkers from structural, microstructural, perfusion, and functional network dimensions. Combining traditional Chinese medicine theories, it explores the points of convergence between traditional Chinese medicine and modern heart-brain axis research, and further analyzes possible pathways for integrating traditional Chinese medicine with modern medicine with the support of neuroimaging, aiming to provide more forward looking ideas for constructing new intervention strategies for the holistic treatment of heart and brain.

Nevertheless, However, to move beyond simple epidemiological links and develop targeted diagnostic and treatment strategies, it is necessary to deconstruct the complex biological interactions that drive the heart-brain axis. The development of cognitive decline from coronary artery disease is not mediated by a single factor, but rather by the combined effects of three pathological factors: hemodynamic failure, systemic microvascular structural damage, and a neuroinflammatory cascade. As we will elaborate in the next section, these mechanisms, ranging from chronic cerebral hypoperfusion to oxidative stress, do not exist in isolation. Rather, they collectively form the basis of vascular disease and generate specific biological targets that underpin the brain structural and functional features subsequently detectable through neuroimaging.

## 2 Literature search and selection methodology

### 2.1 Data sources and retrieval strategies

In order to ensure the scientific rigor and comprehensiveness of this systematic review, we conducted a comprehensive search of major databases, including PubMed, Web of Science, and Embase, and reviewed relevant original studies cited in other reviews. The search strategy integrated medical keywords (MeSH) and free text keywords related to coronary artery disease, cognitive impairment and neuroimaging, including but not limited to: “coronary artery disease,” “cardiovascular disease,” “cognitive impairment,” “dementia,” “heart-brain axis,” “brain-heart interaction,” “magnetic resonance imaging,” “functional magnetic resonance imaging,” “diffusion tensor imaging” and “cerebrovascular disease.” We also included keywords related to traditional Chinese medicine, such as “traditional Chinese medicine,” “traditional Chinese medicine constitution,” and “heart governing spirit,” to identify relevant comprehensive studies. Each group of keywords is combined with Boolean logic “AND” and “OR” to form a system search formula. All searches are in English and cover the database established as of 12 October 2024. In order to avoid omissions, we also manually supplement the search by tracking the references included in this review to screen the original studies that may be eligible, thereby further ensuring the comprehensiveness and systematicness of the literature search.

### 2.2 Inclusion and exclusion criteria

Inclusion criteria: Original research papers, cohort studies, randomized controlled trials, and high-quality English-language review articles that explore the association, mechanisms, or neuroimaging features of CAD-related CI. Exclusion criteria: Only studies specifically targeting non-cardiovascular and non-neurological diseases, animal models lacking translational medicine significance, case reports, conference abstracts, and papers not including neuroimaging or cognitive outcome data.

## 3 Pathophysiological mechanisms of CAD related CI

### 3.1 CI caused by cardiac factors

Cognitive impairment observed in patients with CAD can be partly attributed to cardiogenic factors, namely the direct effects of cardiac dysfunction and the shared pathological mechanisms underlying both conditions (20). Heart failure, a common complication of CAD, can result in chronic cerebral hypoperfusion, and sustained reductions in cerebral blood flow may induce cognitive decline (21). A review has noted that hypoperfusion is one of the major risk factors for cognitive deterioration following impaired cardiac function, while immune responses and oxidative stress play central roles in this process, involving multiple pathways such as disruption of blood brain barrier integrity, glial cell activation, and amyloid deposition (4). Other heart disorders, such as atrial fibrillation or coronary heart disease, may also be linked to CI, with heart failure and neuroinflammation associated with the brain, making cognitive problems even worse by triggering inflammatory cytokine pathways in the central nervous system (7, 22). Large scale clinical studies have further shown that various cardiac diseases including atrial fibrillation and congestive heart failure are significantly associated with cognitive decline, with potential mechanisms encompassing vascular remodeling, cardiogenic microembolism, and chronic cerebral hypoperfusion, all of which ultimately converge to impair cerebral blood supply and reduce cognitive capacity (23).

### 3.2 Association between cerebral small vessel disease (CSVD) and coronary microcirculatory dysfunction (CMD)

Coronary microcirculatory dysfunction is common among patients with CAD and is significantly associated with CSVD, with both conditions jointly contributing to the onset and progression of cognitive impairment (24). A prospective study found that CMD is highly prevalent in individuals with CAD and strongly correlated with CSVD, an association reflected in cerebral hemodynamic abnormalities and declines in cognitive test performance, thereby supporting the hypothesis of a shared microvascular dysfunction mechanism between the heart and brain (24). CMD and CSVD are pathophysiological features that can be interpreted as external manifestations of microvascular arteriopathy: CMD in the heart may cause angina, and CSVD in the brain is linked to the risk of dementia and ischemic stroke due to shared vascular anatomy and associated pathogenic risk factors (25, 26). There is also evidence that CMD and CSVD share multisystem pathophysiology, such as inflammatory responses and vascular regulation, which, when combined, increase cognitive decline (23). It has been shown that coronary atherosclerosis, a primary sign of CAD, is associated with CSVD, suggesting that microvascular dysfunction may be a bridge between heart and brain pathology and potentially significantly influence long-term cognitive prognosis (27). CMD also plays an important role in various conditions such as non-obstructive CAD and heart failure, with its central mechanism involving dysregulation of coronary circulatory blood flow, thereby further reinforcing its pathological interaction with CSVD (28).

### 3.3 Mechanisms of neuroinflammation and oxidative stress

Chronic inflammation and oxidative stress constitute key molecular mechanisms underlying CAD related brain injury (29). Patients with CAD often exhibit persistently elevated inflammatory markers such as CRP, IL 6, and TNF α, and peripheral inflammation can enter the central nervous system through a compromised blood brain barrier or neural pathways, driving long term activation of microglia and astrocytes (30–33). Activated glial cells secrete large amounts of inflammatory mediators and diminish neurotrophic support, progressively impairing synaptic structure and neuronal function; related studies suggest that neuroinflammation and neurodegenerative processes mutually reinforce each other, rendering brain tissue more susceptible to structural and functional alterations under chronic cardiovascular stress (34, 35). Oxidative stress further amplifies inflammatory effects. Ischemia and metabolic abnormalities associated with CAD can markedly increase ROS production and weaken antioxidant defenses, leading to damage of cell membranes and mitochondria and promoting neuronal apoptosis (33, 36, 37). Some studies indicate that dietary and lifestyle factors may improve heart brain health to some extent by modulating oxidative stress and inflammation related gene expression, providing potential intervention targets for the comprehensive management of CAD patients (38).

### 3.4 Cumulative effects of vascular risk factors

Vascular risk factors are a significant factor in the initiation and progression of CAD CI by multiple pathways leading to pathological damage in the neurovascular unit (39). Common vascular risk factors such as hyperglycemia, systemic inflammation, and chronic kidney disease (CKD) can influence brain health through both atrophy-related and non-atrophy-related mechanisms (4, 40, 41). Evidence shows that although no single neuroimaging biomarker can fully capture the complexity of these processes, the combined burden of multiple risk factors borne by an individual can nonetheless robustly predict worsening cognitive outcomes (41). Feng et al. (42) reported that in patients with CAD, glycemic characteristics such as persistent hyperglycemia are significantly associated with mild cognitive impairment (MCI), and this association is strongly moderated by inflammation. In a physiological environment of excessive inflammation, the patient’s disease progression showed a sharp increase in cognitive vulnerability; this observation led us to identify inflammation as a necessary mechanistic hub. It effectively links metabolic disorders with cognitive impairment.

**FIGURE 1:**
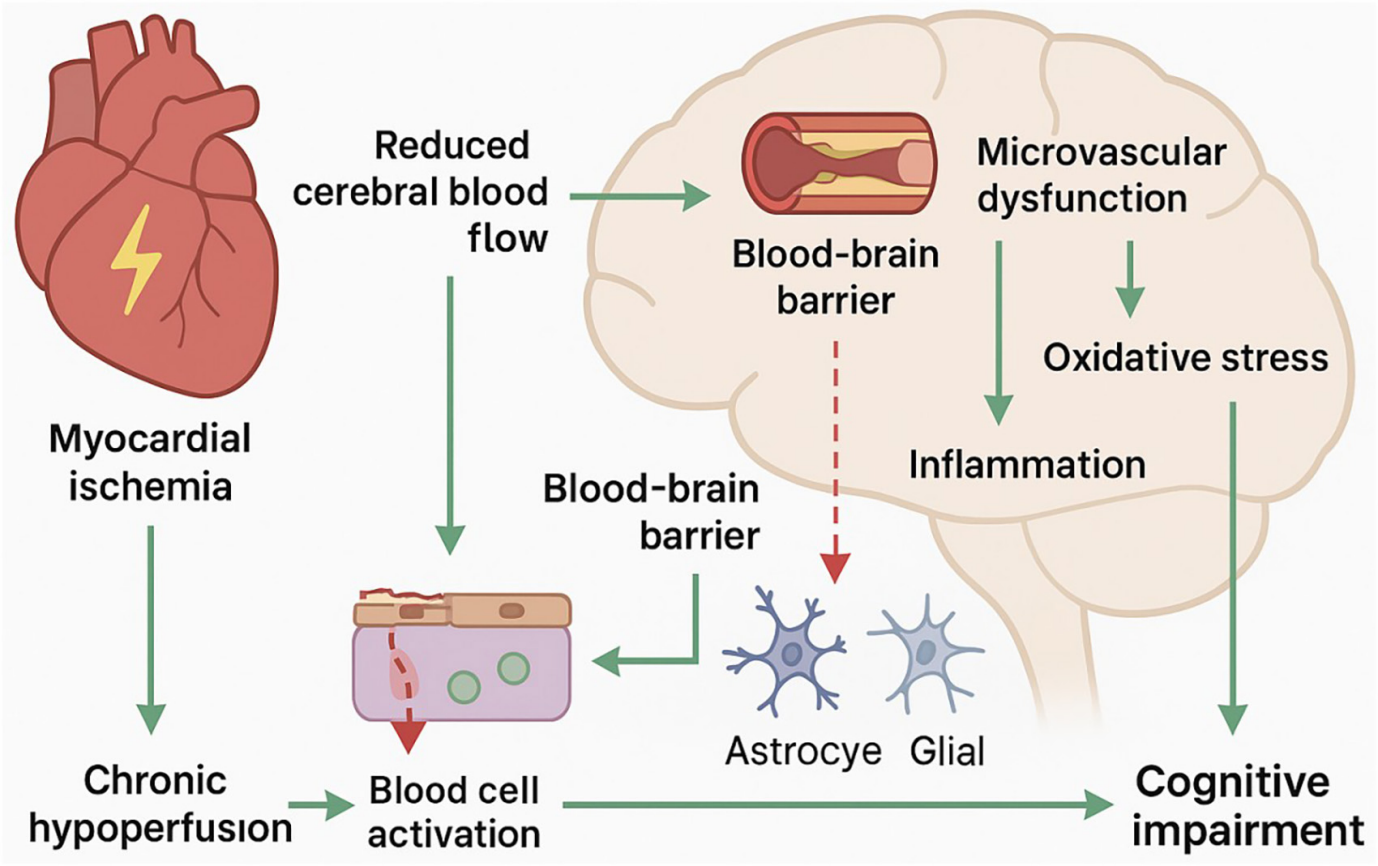
Key pathophysiological mechanisms of coronary artery disease (CAD) related cognitive impairment. The diagram displays the cumulative impact of multiple vascular risk factors on heart-brain axis. This is due to excessive myocardial oxygen supply and demand due to systemic hemodynamic stress. Hypertension, hyperglycemia, CKD and gut microbiota dysbiosis are major factors. They turn out to produce systemic inflammation and oxidative stress due to BBB disruption and neurovascular unit damage. Such a positive feedback effects the pathological process from cardiovascular disease to cognitive impairment.

High blood pressure and diabetes are common arterial risk factors in people with heart disease. They can lead to significant, cumulative effects on cognitive function, either directly impairing cardiovascular function or indirectly by blocking the BBB (1). Metabolism plays a role in CAD-related processes; e.g., CKD can accelerate atherosclerosis through a chronic inflammatory response and altered mineral metabolism, and these CKD-related pathways play an important role in driving cognitive deterioration as renal function declines (43). In CAD patients, the composition and interactions of the gut microbiota are also significantly altered through interactions with host blood metabolites, and this can contribute to the combination of the above risk factors leading to CAD and CI (44). Ultimately, all these processes can be traced back to the underlying imbalance of myocardial oxygen supply and demand. Myocardial ischemia initiates heart problems, causing an oxygen deficiency and therefore the blood stress in heart and cerebral tissues. Due to this, multiple vascular risk factors induce a feedback loop that gradually increases the eventual CI (45) (Figure 1).

## 4 Advances in brain MRI technology in CAD related CI research

Dealing with oxidative stress, inflammation, and microcirculatory dysfunction is global and microscopic, but has macroscopic impacts on brain tissues, hemodynamics, and functional patterns. MRI, an essential translational link, makes such silent microscopic biological processes measurable imaging biomarkers. By employing specific pulse sequences sensitive to proton density, water diffusion rate, and oxygen saturation, modern MRI protocols enable non-invasive mapping of CAD-induced brain injury trajectories, converting abstract concepts such as neuroinflammation and hypoperfusion into observable indicators, including changes in anisotropy fractions, reduced cerebral blood flow, and disruption of functional connectivity networks.

### 4.1 MRI physiological basis of CAD related brain changes

Coronary artery disease-related CI is also developed when the brain’s long-term relationships between heart dysfunction, systemic atherosclerosis and cerebrovascular problems can be observed. Brain MRI offers a multilayered imaging window for this step. Physiologically, the effects of CAD on the brain only include the negative effects of insufficient cerebral perfusion, cerebral small-vessel disease, and desynchronization of large-scale functional networks (38, 46, 47).

Chronic cerebral hypoperfusion is a vital bridge between poor heart function and cognitive damage. In CAD and comorbid heart failure patients often exhibit lower heart output and blood pressure fluctuations, lower cerebral perfusion reserve, and poor autoregulatory capacity. MRI perfusion experiments showed that, in patients with cardiovascular disease, localized cerebral blood flow in key cognitive regions, such as the frontal and parietal lobes and the hippocampus, is dramatically reduced, with a significant effect on executive function, attention, and memory tests (48–50). These results suggest that, even in the absence of brain atrophy or clinical stroke, a mild decline in cerebral perfusion cannot disrupt neuronal energy metabolism or synaptic plasticity, which are initial physiological substrates of CAD symptoms (51, 52).

White matter damage and cerebral small vessel disease (CSVD) are essential morphological markers of CAD-related cognitive impairment. On sMRI, CSVD may manifest as white matter hyperintensities, lacunar lesions, and microbleeds, and is associated with atherosclerosis, endothelial dysfunction, and chronic inflammation (53, 54). It is observed that a larger coronary artery disease burden is associated with larger white matter lesions and larger microstructural abnormalities, which, in turn, lead to slower processing speed and executive function (55, 56). The findings suggest that damage to cerebral white matter and microvasculature largely reflects cerebral manifestations of systemic small-vessel pathology and is a key contributor to CAD-related CI.

Disruptions in large scale functional networks represent a key functional hallmark of CAD related cognitive impairment. Studies have shown that patients with CAD exhibit altered functional connectivity across multiple cognition related networks, including the default mode network, the frontoparietal control network, and the limbic system, characterized by reduced spontaneous activity in certain nodes, weakened network integration, and redistribution of hub regions (6, 12, 57–60). These network abnormalities are strongly associated with global cognitive performance and deficits in specific cognitive domains, suggesting that long term cardiogenic hypoperfusion and CSVD related white matter damage may weaken neuronal synchrony and long range connectivity, ultimately leading to structural and functional reorganization of cognitive networks (26, 61).

**FIGURE 2:**
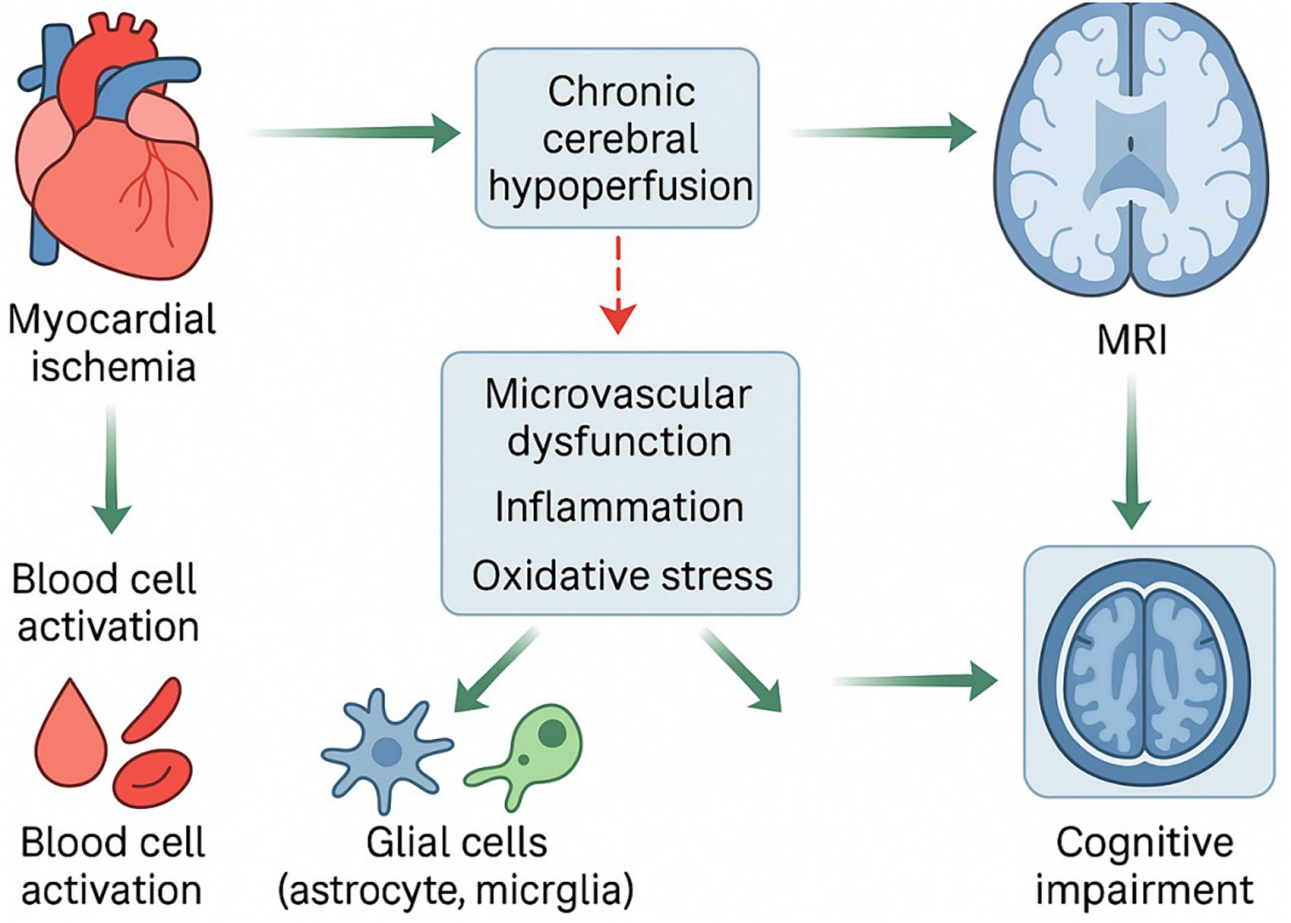
Magnetic resonance imaging (MRI) visible mechanistic pathway of coronary artery disease (CAD) related cognitive impairment. The diagram maps the sequential progression of brain injury along the heart-brain axis as revealed by multimodal MRI. The trajectory begins with cardiac dysfunction causing chronic cerebral hypoperfusion. This hemodynamic failure, combined with inflammatory insults, leads to structural alterations, manifesting as cerebral small vessel disease (CSVD) and white matter microstructural integrity loss. These structural deficits subsequently disrupt the synchronization of large-scale functional networks, ultimately precipitating cognitive decline. This visualization underscores the utility of multimodal imaging in tracking the transition from vascular pathology to neurological symptoms.

Taken together, the continuous sequence of changes revealed by brain MRI ranging from hypoperfusion to small vessel disease and white matter damage, followed by large scale network reorganization constitutes a physiological map of CAD related cognitive impairment at the cerebral level, and provides a clear mechanistic framework for future research employing rs-fMRI, sMRI, and multimodal imaging (Figure 2).

### 4.2 Advances in the application of functional MRI

Resting-state functional MRI (rs-fMRI) has become one of the most widely used functional imaging methods in studies of CAD related CI. Numerous studies have found that patients with coronary heart disease, even in the absence of overt clinical stroke, already exhibit reduced spontaneous activity in regions such as the frontal lobe, parietal lobe, and parahippocampal gyrus, and these abnormal patterns are significantly correlated with scores on scales such as the MoCA and MMSE (54, 55). Further graph theoretical analyses indicate that the functional brain networks of CAD patients show weakened small world properties, reorganization of hub regions, and reduced global efficiency, suggesting that long term cardiovascular burden and microvascular pathology may induce network level topological reconfiguration (61).

Building on this foundation, some studies have begun to incorporate analytic frameworks based on causal connectivity and dynamic connectivity to investigate how CAD affects the temporal architecture of functional brain networks. Hassan et al. (12), using structural equation modeling and Granger causality analysis, found that the directionality of effective connectivity within the frontal parietal limbic circuit is altered in CAD patients, with a particularly notable reduction in the top down regulation exerted by the prefrontal cortex on the hippocampus and precuneus; this finding is considered one of the functional bases for impaired executive function and episodic memory. Moreover, fMRI studies in heart failure cohorts have shown that reduced cardiac output is closely associated with weakened functional connectivity between the DMN and the frontoparietal network, further underscoring the influence of cardiac function on the homeostasis of cognitive networks (47).

Task based fMRI has been relatively underutilized in CAD related research, but small sample studies employing working memory tasks, emotion recognition tasks, or blood pressure reactivity tasks have observed reduced or compensatory overactivation in regions such as the prefrontal cortex, cingulate gyrus, and insula in patients with CAD or heart failure, suggesting that cardiovascular disease may alter the mobilization pattern of task related neural resources (62). With continued refinement of task paradigms and scanning protocols, task based fMRI holds promise for more precisely characterizing the vulnerability of specific cognitive domains such as executive control and social cognition in CAD populations, thereby providing a foundation for individualized cognitive assessment and targeted intervention.

### 4.3 Advances in the application of sMRI

Structural MRI, through volumetric assessment, cortical thickness analysis, and VBM approaches, has provided substantial macroscopic morphological evidence for CAD related CI (12, 63, 64). Early studies in CAD patients undergoing revascularization reported significantly reduced gray matter volumes in the temporal lobe, hippocampus, and frontal lobe compared with healthy controls, and the degree of brain atrophy was associated with the extent of coronary lesions and a history of myocardial infarction (9, 17, 58). Subsequent large sample studies in heart failure further confirmed widespread gray matter volume loss and cortical thinning in chronic heart failure patients, particularly involving key cognitive structures such as the frontal lobe, hippocampus, and cingulate gyrus. These imaging alterations were closely correlated with cognitive test performance and biomarker levels (65–67).

Diffusion tensor imaging (DTI) and other microstructural imaging techniques have played an important role in revealing CAD related white matter damage. Heart failure and CAD patients can show more FA or MD in regions such as the frontal striatal areas, corpus callosum, and inner capsule, which correspond to myelin damage, lower axonal integrity, and thus slower speed, attention, and executive function (68, 69). CSVD studies also show that the severity of white matter hyperintensities and microstructure damage is associated with cardiovascular risk factor burden and coronary atherosclerosis (70, 71). Longitudinal MRI studies in heart failure patients show that gray matter loss and increased white matter microstructure over time parallel declines in heart function and clinical events, highlighting the dynamical nature of heart-brain pathology (72, 73).

### 4.4 Advances in the application of multimodal image synthesis

With the growing scientific evidence of the relationship between CAD and CI, multimodal brain MRI has proven to be a powerful tool for revealing pathological behavior. Multimodal MRI uses structure, function, DTI and perfusion MRI to provide information on brain structure, function and hemodynamics from multiple dimensions, which is superior to single-modal analysis (5). For CAD-related CI, it is easier to study abnormal brain changes and identify potential neural biomarkers to help treat cognitive decline. For example, based on fMRI functional connectivity and topological analysis, combined with the structural changes of sMRI, abnormal activation patterns of brain network regions were found in CAD patients (59, 69). Studies have shown that multimodality may map the changes of low-frequency oscillation activity in CAD patients at rest, which may be related to the decline of cognitive function (5, 58). The current research has developed from early single-modal analysis to multi-modal fusion, thus improving the accuracy of pathological mechanism analysis.

Recent studies have effectively integrated heterogeneous neuroimaging data using advanced machine learning frameworks. Multiple kernel learning (MKL) and support vector machine (SVM) have been successfully applied to predict the risk of cognitive decline in CAD patients. In these models, researchers constructed different “nuclei” to characterize structural and functional features, respectively: one nucleus processed anatomical features extracted from sMRI to capture cumulative neuronal loss driven by chronic hypoperfusion; another kernel processes hemodynamic features extracted from resting-state functional magnetic resonance imaging to detect real-time interruptions in neural network synchronization. By mathematically weighting and fusing these kernels, the model utilizes complementary information about structural atrophy and dysfunction (74). The study of the vascular cognitive impairment cohort showed that this multimodal fusion method has higher diagnostic sensitivity than the single-modal model, and can effectively identify high-risk patients in the early stage, and the single imaging biomarkers of these patients may still be ambiguous (75).

In multimodal applications, experimental designs primarily include prospective cohort analyses and case control studies comparing brain imaging characteristics between healthy controls and patients with CAD. One study, combining diffusion kurtosis imaging (DKI) and functional MRI, identified significant associations between microstructural alterations and cognitive performance in 28 CAD patients, with these changes interpreted as the neural substrates of cognitive impairment (68). Similarly, other work integrating rs-fMRI and sMRI observed abnormal topological properties of functional connectivity networks in patients with CHD, characterized by reduced global efficiency, which was associated with deficits in executive function and memory (61). At the same time, multimodal MRI can also provide benefits in hemodynamic measurement: for instance, perfusion imaging can measure cerebral blood flow in healthy ICAS patients. These changes may increase the risk of cognitive decline and lead to refinement of treatment decisions (5). These results not only confirmed that the network connection problem is a key component of CAD-related CI, but also studied the mechanism of cardiogenic dementia and focused on the reduction of blood flow in support function.

## 5 Advances in traditional Chinese medicine and the heart-brain axis in CAD related CI research

The evolving accuracy of multimodal MRI - especially when used to unravel the complexity of large-scale functional networks and neurovascular coupling - not only provides diagnostic clarity; it constructs an empirical scaffold for the ancient and systematic framework of traditional Chinese medicine. Western pathology is localized. In contrast, the network-based rs-fMRI mental temperament resonates with the overall epistemology of traditional Chinese medicine, which can be said to have amazing fidelity. Take the “heart-brain axis.” This concept in modern neurocardiology has a basic functional syntax with the classic doctrine of “heart governing mind” (spirit/mind). Then, neuroimaging goes beyond the role of a simple lesion capturer. It becomes a validation engine. By reinterpreting “Qi” and “Blood” through the physiological lens of functional connectivity and cerebral perfusion, it effectively grounds TCM’s systemic strategies in quantifiable science.

### 5.1 Holistic view of traditional Chinese medicine and the heart-brain axis

The holistic thinking of TCM originates in the Yin-Yang and Five Elements theories, in which the internal organs are connected and coordinated through blood flow (e.g., meridians or qi-blood flow). Based on this principle, TCM emphasizes the heart-brain connection. For example, heart disease can interfere with heart function, but cardiac disease can also exert feedback on the brain (76–78). This is reflected in TCM’s multi-target multi-pathway treatment. For instance, Niu et al. (78) demonstrated that the bidirectional effects of heart and brain functions could be controlled through related organs (e.g., liver, spleen, kidney), consistent with TCM theories (echog, liver-brain mutual help, brain-spleen association, brain lip linkage, brain/kidney coordination). The TCM holistic paradigm also aligns with modern network pharmacology, which studies biological networks from a systems perspective and further supports TCM’s integrative therapeutic benefits in complex diseases such as CAD-related CI (79).

The heart-brain axis refers to the bidirectional interactions between the heart and the brain. It is a well-known theory in TCM. In TCM, the heart governs shen, so that cardiac disorders can cause brain dysfunction, and this can result in pathological heart disorders, which is also in good agreement with modern medical understanding of the heart-brain axis (80). It has been shown that TCM can guide clinical practice with the concept of heart brain interconnection, using theories such as Five Zang Organ connection between the liver and heart, and kidney brain axis theory, where the kidney governs bones and generates marrow, so that organs including liver, kidney, intestine keep the heart-brain axis in balance (78, 81, 82). In CAD-related CI, heart-brain imbalance is characterized by vascular decline and an inflammatory cascade, processes that TCFM wants to control through multitasking. For example, TCM in the gut-brain axis views the gut microbiota as a key driver of heart and cognitive function, and it can be used to achieve multi-pathway treatments aimed at both cardiovascular health and cognitive performance (83, 84).

### 5.2 The influence of traditional Chinese medicine constitution on CAD related CI

Traditional Chinese medicine constitution, which categorizes individuals based on their physical and metabolic characteristics, plays a crucial role in susceptibility to cognitive impairment. The Constitution in Chinese Medicine Questionnaire (CCMQ) has been used to establish baseline constitution types in older populations. The study using MMSE and MoCA showed that some physical factors, such as qi deficiency or blood stasis, were not well-correlated with neurocognitive scores. Therefore, they may be associated with mood or metabolic disorders (85). Some physical types, such as phlegm-dampness and yin-deficiency, may damage specific cognitive subdomains, including executive function and delayed recall (86).

Recent neuroimaging studies are bridging the gap between these physical concepts and the visualization of brain pathology. Modern diagnosis mainly depends on subjective signs. Modern MRI suggests that different TCM constitutions may be related to different brain structures and functional attributes (Table 1). For example, recent voxel-based morphometry (VBM) studies have shown that patients with yang-deficiency constitution (usually metabolic dysfunction) have smaller gray matter volumes in the hippocampus and temporal lobe, which is related to the atrophic contour of vascular cognitive impairment (87). At the same time, resting-state functional magnetic resonance imaging showed that patients with qi-stagnation constitution showed changes in the functional connectivity of the prefrontal-limbic circuit, providing a neural basis for the anxiety and cognitive stiffness that often occur in these patients (88).

**TABLE 1:** Integrated associations between traditional Chinese medicine (TCM) constitutions, neuroimaging biomarkers, and cognitive phenotypes.

TCM constitution	Key pathophysiological mechanism	Associated cognitive deficits	Primary MRI neuroimaging biomarkers
Yang deficiency (YADC)	Metabolic hypofunction; impaired thermogenesis; vascular dysregulation; Immune suppression; mitochondrial dysfunction.	Global cognitive decline; memory deficits; executive dysfunction.	sMRI: reduced gray matter volume (GMV). Atrophy in hippocampus (total, CA1, molecular layer), Subiculum, Presubiculum, and Fimbria.
Phlegm-dampness (PDC)	Lipid metabolism disorders; systemic inflammation; insulin resistance; gut microbiota dysbiosis (Firmicutes/Bacteroidetes ratio).	Executive function impairment; processing speed decline; visuospatial deficits.	sMRI: increased white matter hyperintensities (WMH); lacunar infarcts. DTI: reduced fractional anisotropy (FA) in frontal-subcortical tracts.
Blood stasis (BSC)	Hemorheological abnormalities; microcirculatory stasis; endothelial dysfunction; high thrombosis risk; Impaired glymphatic clearance.	Vascular dementia features; executive dysfunction; slower reaction times.	DTI: widespread loss of white matter integrity (reduced FA, increased MD/RD/AD). Disrupted structural connectivity in projection fibers (internal capsule).
Qi stagnation (QSC)	Emotional dysregulation (depression/anxiety); autonomic imbalance; HPA axis dysfunction; altered neurotransmitter levels (5-HT, DA).	Attentional deficits; emotional memory bias; reduced cognitive flexibility.	fMRI: altered functional connectivity (FC) in default mode network (DMN) and Frontoparietal Network. Dysregulation in prefrontal-limbic circuits (Amygdala-PFC uncoupling).
Qi deficiency (QDC)	Reduced cardiac output; chronic cerebral hypoperfusion; mitochondrial dysfunction; energy metabolism deficit.	Fatigue-related cognitive decline; general cognitive slowing; attention deficits.	ASL MRI: reduced cerebral blood flow (CBF) (hypoperfusion), esp. in frontal/parietal lobes. rs-fMRI: decreased amplitude of low-frequency fluctuations (ALFF).

In CAD, qi deficiency and blood stasis also play an important role. Pathologically, this component is associated with modern mechanisms of chronic cerebral hypoperfusion and microvascular endothelial dysfunction. MRI studies using ASL showed reduced cerebral blood flow (CBF) in these patients, and DTI often showed impaired white matter integrity in the frontal-subcortical pathway (89, 90). These findings mean that TCM constitution is not only a theory, but also related to the physical and measurable changes of cerebrovascular and neural structures.

### 5.3 The effect of TCM intervention on the regulation of the heart-brain axis on CAD related CI

In recent years, with the in-depth application of neuroimaging technology in clinical trials, the evaluation system of the efficacy of traditional Chinese medicine in the prevention and treatment of cardiovascular and cerebrovascular comorbidities is undergoing a qualitative leap. A randomized controlled trial specifically included 200 patients with stable angina pectoris and CI. The results showed that after 8 weeks of STDP treatment on the basis of routine Western medicine treatment, the patients’ Montreal Cognitive Assessment (MoCA) scores were significantly improved compared with the control group. At the same time, the scores of the Neuropsychiatric Questionnaire (NPI) and TCM syndrome scores also decreased significantly. This study provides direct clinical evidence that STDP can not only relieve angina symptoms, but also substantially improve the overall cognitive level of CAD patients by improving the microcirculation of the heart and brain (91). Lan et al. (92) used rs- fMRI to evaluate the effect of acupuncture on brain function in CSAP patients and found that acupuncture treatment not only significantly reduced the frequency of angina attacks, but also specifically modulated the low-frequency amplitude (fALFF) of Calcarine and Precuneus. These two regions are key nodes of the brain’s visual processing and default mode network. More importantly, acupuncture repaired the abnormal functional connections (FC) between these cognitive/emotion-related brain regions and the pain matrix. This finding provides a visualized neurobiological explanation for the traditional Chinese medicine concept of “the heart governs the mind,” suggesting that acupuncture improves brain function while alleviating cardiac symptoms by reshaping damaged brain functional networks. Another fMRI study of laser acupuncture at the Neiguan acupoint showed that stimulation of this acupoint specifically modulated the activity of the hypothalamus and brainstem autonomic nuclei. This suggests that acupuncture may improve cardiac and cerebral perfusion by reflexively modulating autonomic tone of the heart and cerebral blood vessels through the central nervous system (93).

There is growing evidence that traditional exercises such as Tai Chi and Baduanjin can improve cognition in patients with heart failure and CAD. Systematic reviews and meta-analyses have shown that traditional Chinese mind-body exercises can significantly improve MoCA and MMSE scores in elderly high-risk cardiovascular patients. In patients with heart failure, the Tai Chi training group showed better cognitive function preservation than the routine care group, which may be related to its improved exercise tolerance, reduced systemic inflammation and relief of psychological stress (94). Although there are few MRI studies specifically for CAD patients, long-term Tai Chi practice has been shown to increase hippocampal gray matter volume and enhance functional connectivity of the prefrontal cortex in a broad elderly population (95). This suggests that mind-body exercises may induce structural neuroplasticity changes in the brain through a dual mechanism of aerobic exercise and complex motor cognitive training.

Nevertheless, it should be emphasized that direct, high-quality neuroimaging evidence specifically demonstrating the effects of TCM interventions on brain MRI metrics or cognitive networks in CAD populations remains limited. Current findings are largely derived from small-scale studies, non-CAD cohorts, or indirect cognitive assessments. Therefore, assertions regarding the neuroprotective effects of TCM in CAD-related cognitive impairment should be regarded as hypothesis-generating rather than definitive conclusions. Future large-scale controlled trials, including standardized cognitive tests and multimodal brain MRI, are needed to validate these preliminary observations and elucidate the neuroimaging mechanisms of TCM-mediated heart-brain regulation.

## 6 Future research directions

The appeal of integrating traditional Chinese medicine interventions into the cardiovascular system is undeniable. However, due to the flexibility of interpretation of syndrome differentiation and treatment and the lack of quantifiable biological indicators, translating it into standardized clinical practice still faces challenges. Therefore, in order to translate potential into rigorous precision, future research must actively shift toward a data-centric epistemology. Observational correlations alone are far from sufficient. Combining high-field magnetic resonance imaging, computational intelligence, and multi-omics technologies can make the subjective symptoms that doctors traditionally observe objective and measurable, and transform those vague symptom manifestations into specific, quantifiable brain imaging indicators. This helps to construct a unified diagnostic framework that reconciles the overall logic of traditional practices with the precision of modern technologies. Therefore, we have outlined the following development path:

### 6.1 Multimodal omics-imaging-cognition translational framework

To address the unclear biological mechanisms of CAD-CI, future research should employ an integrated omics-imaging framework. A practical model should combine multimodal brain MRI with circulating inflammatory markers, endothelial function indicators, and, where feasible, transcriptomics or metabolomics analysis. Machine learning-based data fusion methods can be used to map molecular features onto imaging phenotypes, linking systemic inflammatory burden with white matter network disruption or inadequate perfusion. This approach can construct a gene-molecule-imaging-cognition causal chain, thereby identifying imaging biomarkers with a biological basis, clinical interpretability, and clearly defined mechanisms.

### 6.2 MRI-guided objectification of TCM syndromes

Using neuroimaging to objectively validate the differentiation of TCM syndromes is one of the most promising but not yet fully developed research directions. Future studies may adopt a stratified cohort design to group patients with CAD based on standardized TCM constitution or syndrome scores, which can be assessed using validated tools such as the CCMQ.

Within each subgroup defined by TCM syndromes, brain MRI data can be quantitatively compared. These data include cerebral blood flow measured by ASL, white matter integrity assessed by DTI, and functional connectivity within the affective–cognitive network. Brain imaging findings can be followed over time, before and after specific TCM treatments. Changes in syndrome scores and cognitive test results can be observed simultaneously. In this way, brain imaging provides a direct link between TCM descriptions and modern brain science. Syndrome identification can shift from a primarily subjective judgment to features that can be measured and replicated using neuroimaging.

### 6.3 Development of personalized precision medicine for cardiovascular and cerebrovascular diseases

Mapping together MRI, clinical cardiology, and TCM assessment has provided us with better personalized risk stratification. Further, future work will need comprehensive prediction models that simultaneously incorporate both imaging-derived brain vulnerability patterns with cardiovascular risk features and TCMR syndrome. In practice, a multi-level risk scoring system needs to be developed that predicts high-risk coronary artery disease patients with a rapid decline in cognitive function and determines the reasoning behind personalized intervention. From cardiovascular health to TCM syndromes, this precision medicine can bring benefits both in cardiovascular prognosis and long-term cognitive function.

## 7 Summary

Increasing epidemiological, mechanistic, and neuroimaging evidence has shown that CAD and CI have standard pathophysiology processes rather than independent comorbidities. Chronic cerebral hypoperfusion, microvascular dysfunction, systemic inflammation, oxidative stress, and cumulative risk interact with the neurovascular unit and large-scale brain network, leading to progressive cognitive decline.

Multimodal brain MRI is an integrated perspective of these heart–brain interactions by translating complex multisystem pathology to measurable structural, perfusion, and functional network biomarkers. MRI serves as a bridge between modern heart-brain axis research and classic Chinese medicine that has empirical support for integrated notions such as systemic control and heart-bios relationship. This imaging-based approach is of great interest to future research and clinical control of heart-Bios related diseases, by earlier risk stratification, mechanism assessment, and objective assessment of comprehensive treatment strategies for heart-biology.
